# Newborn Screening Quality Assurance Program for CFTR Mutation Detection and Gene Sequencing to Identify Cystic Fibrosis

**DOI:** 10.1177/2326409816661358

**Published:** 2016-08-01

**Authors:** Miyono M. Hendrix, Stephanie L. Foster, Suzanne K. Cordovado

**Affiliations:** 1 Centers for Disease Control and Prevention, Atlanta, GA, USA

**Keywords:** cystic fibrosis, *CFTR*, mutation, newborn screening, next-generation sequencing

## Abstract

All newborn screening laboratories in the United States and many worldwide screen for cystic fibrosis. Most laboratories use a second-tier genotyping assay to identify a panel of mutations in the CF transmembrane regulator (*CFTR*) gene. Centers for Disease Control and Prevention’s Newborn Screening Quality Assurance Program houses a dried blood spot repository of samples containing *CFTR* mutations to assist newborn screening laboratories and ensure high-quality mutation detection in a high-throughput environment. Recently, *CFTR* mutation detection has increased in complexity with expanded genotyping panels and gene sequencing. To accommodate the growing quality assurance needs, the repository samples were characterized with several multiplex genotyping methods, Sanger sequencing, and 3 next-generation sequencing assays using a high-throughput, low-concentration DNA extraction method. The samples performed well in all of the assays, providing newborn screening laboratories with a resource for complex *CFTR* mutation detection and next-generation sequencing as they transition to new methods.

## Introduction

Cystic fibrosis (CF) is one of the most common autosomal recessive disorders that affects approximately 1:4000 people of Western European, North American, and Australasian descent. When CF is identified and treated early, patients avoid many of the devastating clinical consequences, allowing for improved growth, reduced hospitalizations, and longer life span, which resulted in the US Centers for Disease Control and Prevention (CDC) recommending that CF be included in newborn screening panels in the United States.^1,2^ Newborn screening for CF begins with an immunoassay that measures the pancreatic enzyme immunoreactive trypsinogen (IRT), which is elevated in newborns affected with CF.^3,4^ Since IRT can be elevated for reasons other than CF, this test alone does not have the specificity required for newborn screening. In 1989, scientists discovered the CF transmembrane regulator (*CFTR*) gene on chromosome 7.^5^ Defects in the *CFTR* gene that alter structure, function, or expression of this protein can lead to malfunctions or disease processes in the lungs, upper respiratory tract, gastrointestinal tract, pancreas, liver, sweat glands, and genitourinary tract.^6^

All US states and many international programs have implemented routine newborn screening for CF. Most US programs use an algorithm that involves at least 1 initial measurement of IRT from a dried blood specimen (DBS) taken from all newborns and then testing for a panel of *CFTR* mutations on a subset of babies with elevated IRT.^7,8^ The panel of *CFTR* mutations can be variable between programs but typically includes the American College of Medical Genetics (ACMG) recommended 23 mutations and often additional mutations.^7–10^ Newborns with either 1 or 2 *CFTR* mutations are considered screen positive by most programs and are sent to CF care centers for diagnostic workup. Although this algorithm has a relatively low false-negative rate, it has quite a high false-positive rate with most babies being carriers of 1 *CFTR* mutation and do not display any symptoms associated with CF. As an example, the state of Wisconsin reported a false-positive rate as high as a 90%^11^ and the New York state found as high as a 94% false-positive rate (excludes screen positives with no mutations identified).^12^ The false-positive rates vary between programs most often because of the differences in the IRT cutoff but sometimes because of the mutation panels used. Currently, the only US program that identifies a screen positive as 2 identified *CFTR* mutations is the state of California, which initially tests for a panel of *CFTR* mutations in babies with elevated IRT. If a newborn has only 1 mutation from the California panel, the *CFTR* gene is sequenced and only those babies with 2 mutations are sent for clinical evaluation. Using this algorithm, California reported that 34% of their screen-positive newborns were CF, 53% had a milder form of *CFTR*-related metabolic syndrome (CRMS), and 13% were carriers with complex mutations.^13^

False CF-positive results, while unavoidable in newborn screening, cause parental anxiety, unnecessary clinical testing, and downstream genetic counseling.^14–17^ Thus, there are ongoing efforts to redefine a positive newborn screening test such that it requires the identification of 2 CF-causing *CFTR* mutations^18^ similar to what is being done in California.^13^ Since there are >2000 mutations or variants in the *CFTR* gene with more still being discovered,^19,20^ the requirement of identifying 2 CF-causing mutations would likely necessitate either gene sequencing or a greatly expanded genotyping panel of *CFTR* mutations. As the complexity of *CFTR* mutation detection in newborn screening expands, there is a need for more extensively characterized dried blood spot quality assurance materials to ensure that high levels of accuracy are maintained in these analytical measurements.

The CDC’s Newborn Screening Quality Assurance Program (NSQAP) provides DBS proficiency testing to United States and international laboratories for both IRT (N = 215 laboratories, quarter 1 of 2016) and *CFTR* mutation detection (N = 68 laboratories, quarter 1 of 2016).^21,22^ The NSQAP’s CF DNA DBS repository, made from CF patient and family blood samples, contains a wide variety of *CFTR* mutations including the 23 recommended by ACMG as well as 47 additional mutations. Each repository sample is characterized extensively by CDC’s Molecular Quality Improvement Program Laboratories using Sanger sequencing and commonly used genotyping methods to ensure robust performance in newborn screening laboratories. As the complexity of CF molecular methods continues to evolve, CDC has performed a comprehensive evaluation and characterization of the CF DNA DBS repository samples using a diverse array of genotyping and next-generation sequencing methods that would be amenable in the newborn screening laboratory environment.

## Materials and Methods

### Samples

Samples from 198 patients and family members affected by CF were collected from CF care centers located in Maryland, Ohio, and Wisconsin and more recently in California in collaboration with the Sequoia Foundation and the California Department of Public Health. All blood was collected in EDTA blood collection tubes from adult donors with at least 1 *CFTR* mutation (Becton Dickinson, Franklin Lakes, New Jersey) and shipped to CDC, where blood was spotted on to Whatman 903 filter paper (Piscataway, New Jersey) to create dried blood spots (75 µL) for quality assurance. This project was approved by the institutional review boards of all participating CF care centers, and the CDC’s Office of Science at the National Center for Environmental Health determined that CDC was not involved with human subjects under 45 CFR 66.012(d).b because all specimens are deidentified and cannot be traced back to the donor.

### Newborn Screening Quality Assurance Program CF DNA Proficiency Testing (PT) Program

Each participating laboratory received 5 blind-coded proficiency testing specimens 4 times a year, and laboratories reported both the genotyping results and clinical assessments to CDC. These results were evaluated based on the program’s stated mutation panel and molecular algorithm. Programs were informed to assume all samples have an elevated IRT that would trigger their algorithm to test for *CFTR* mutations. To ensure accurate grading, each programs provided descriptive information including *CFTR* genotyping/sequencing method, mutation detected or exons sequenced if not using a commercial method, secondary/confirmatory method, description of when and how a secondary/confirmatory method is used, and DNA extraction method.^23^

### DNA Extraction and Quantitation

Genomic DNA was extracted from 250-µL whole blood (EDTA anticoagulant) using the Qiagen QIACube Micro spin columns and resuspended in 100 µL of Tris-buffered EDTA (Valencia, California). The DNA was quantified using the NanoDrop spectrophotometer (Wilmington, Delaware) and diluted to 10 ng/µL for direct use only in Sanger sequencing. Genomic DNA was also extracted from one 3.2-mm DBS punch using the Qiagen Generation DNA Purification and Elution Solutions. The punch was washed 2 times for 15 minutes in 150 µL of DNA Purification Solution followed by one 15-minute wash with 150 µL of DNA Elution Solution. All washes were performed at room temperature with slight agitation. Genomic DNA was eluted from the punch in 50 µL of DNA Elution Solution after incubating at 99°C for 15 minutes. All assays other than Sanger sequencing used DNA extracted from DBS. When quantification of DNA extracted from DBS punches was required, a real-time polymerase chain reaction (PCR) of the *RNase P* gene using the TaqMan RNaseP Control Reagents was used (Thermo Fisher Scientific, Waltham, Massachusetts). The standard curve was made from human genomic DNA (Roche Applied Science, Penzberg, Germany).

### CFTR Genotyping

Following the manufacturer’ instructions, the NSQAP samples were genotyped and analyzed using 3 commercially available products: InPlex CF Molecular Test 40+4 (Hologic, Marlborough, Massachusetts), xTAG CF39v2 and CF60v2 kits (Luminex, Austin, Texas), and the MiSeqDx CF 139-variant assay (Illumina, San Diego, California). The InPlex CF Molecular test 40+4 followed the manufacturer’s instruction for the in vitro diagnostic (IVD) InPlex CF Molecular Test IVD with 1 modification—the second cycling step during the amplification process was increased from 12 to 14 cycles. The DNA volume used for the Hologic InPlex CF Molecular Test 40+4, both the Luminex xTAG 39 and 60 kits, and the MiSeqDx CF 139-variant assay kit was 5 mL of the Generation extraction’s 50 mL total volume (5–10 ng DNA). Samples were also analyzed using 45 unique TaqMan *CFTR* single-nucleotide polymorphism (SNP) assays that include all but the I148T (c.443T>C) and D1270N (c.3808G>A) mutations in the InPlex CF Molecular Test 40+4 assay and the addition of the I506V (c.1516A>G) and I507V (c.1519A>G) variants. Each 10 µL reaction consisted of 1× DurAmp v2 Master Mix (Thermo Fisher Scientific), 1 × of SNP genotyping probe mix, and 1 µL of the Generation extraction’s 50 µL total volume (1–2 ng DNA). All run data were loaded into Thermo Fisher Scientific’s Genotyper software and analyzed with Hardy-Weinberg analysis with noted exceptions. Hardy-Weinberg analysis was disabled for the F508del (c.1521_1523delCTT), I507del (c.1519_1521delATC), I506V (c.1516A>G), I507V (c.1519A>G), F508C (c.1523T>G), 5T (c.1210–12[5]), and 9T (c.1210–12[9]) assays. (Note: since the writing of this manuscript, the Hologic Inplex CF assays have been recalled and discontinued.)

### Next-Generation Sequencing of CFTR

#### Ion AmpliSeq CFTR Panel on the Ion Torrent PGM

Target regions of the *CFTR* gene were amplified in 2 amplicon pools that covered all exons, untranslated regions (UTRs), and regions of interest in introns 12 and 22 and bar-coded using a custom Ion AmpliSeq *CFTR* Panel and IonXpress bar codes (Thermo Fisher Scientific). Each pool required 6 µL of Generation extracted genomic DNA (5–50 ng/pool; average: 18 ng/pool) and was quantitated, pooled, amplified, enriched, and sequenced on a 318 chip according to manufacturer’s instructions. Data from the Ion PGM (Thermo Fisher Scientific) were processed and aligned to the human genome reference sequence (hg19, build GRCh37). In order to determine the level of sequence coverage for the targeted genomic region, the manufacturer provided Coverage Analysis plug-in (v4.0-r73765) was utilized. Variants were called and annotated using the Variant Caller plug-in (v4.0-r73742) with a customized hotspot file consisting of 240 unique variants also provided by the manufacturer. The custom hotspot file is available upon request. The data were visually inspected using Integrative Genomics Viewer (IGV) from the Broad Institute (Cambridge, Massachusetts).^24,25^ All analyzed data from the Ion PGM were then compared for concordance against the Sanger sequence data.

#### Swift Biosciences Accel-Amplicon CFTR Panel for Illumina Platforms

Target regions of the *CFTR* gene were amplified in a single amplicon pool that covered all exons, UTRs, and regions of interest in introns 12 and 22 known to contain mutations 1811 + 1.6kbA>G (c.1679+1.6kbA>G) and 3849+10kbC>T (c.3717+12191C>T) using the Accel-Amplicon *CFTR* Panel for the Illumina MiSeq Platform (Swift Biosciences, Ann Arbor, Michigan). The genomic DNA input was between 10 and 30 ng, and the libraries were prepared according to the manufacturer’s instructions. The libraries were diluted by 1:100 000 and quantified using KAPA Biosciences Library Quantification kit KK4835 (Wilmington, Massachusetts) according to the manufacturer’s recommendation on the QuantStudio 12K Flex (Thermo Fisher Scientific). Libraries were diluted to either 2 or 4 nmol/L and pooled together for denaturing and subsequent loading on to the MiSeq flow cell at a final concentration of 12 to 16 pmol/L. The samples were run on the MiSeqDx instrument research mode using either the MiSeq Reagent Kit v2 Standard (300 cycles) or MiSeq Reagent kit v2 Micro (300 cycles; Illumina).

As an open system, data from the Accel-Amplicon *CFTR* Panel were processed using several freeware bioinformatic tools to create a custom analytical pipeline. The first step was to trim the 5′- and 3′-anchored primers using Cutadapt.^26^ The data were then aligned to the human genome reference sequence (hg19, build GRCh37) using Burrows-Wheeler Aligner BWA-MEM version 0.7.5a–r405,^27^ and variants were extracted using FreeBayes version v1.0.2^28^ and GATK version 3.5 (3.5.0-g36282e4)^29^ using a Browser Extensible Data (BED) file supplied by the manufacturer to limit the sequence area of interest. The data were also visually inspected using IGV. All analyzed data were then compared for concordance against the Sanger sequence data.

### Sanger Sequencing of CFTR

Sanger sequencing was performed for all exons, intron/exon borders, and a region of interest in intron 22 known to contain mutation 3849+10kbC>T (c.3717+12191C>T). The *CFTR* gene was amplified using primer sets (RSS000010013) described in the National Center for Biotechnology Information’s Probe database. Each region was amplified from 5 to 10 ng of genomic DNA in a 10 µL reaction, using 10 pmol each of forward and reverse primers in the RSS000010013 primer sets, and 1 × HotStarTaq Master Mix (Qiagen, Valencia, California). Cycling conditions were as follows: 10-minute denaturing step at 95°C; 40 cycles at 95°C for 30 seconds, 62°C for 30 seconds, and 72° C for 1 minute; 10-minute extension at 72°C, followed by a 4°C hold. Unused primers and nucleotides were removed using ExoSAP-IT (Affymetrix, Santa Clara, CA), and sequencing was performed using BigDye Terminator Ready Reaction kit, version 1.1. The cycle sequencing reaction consisted of 1 µL of BigDye Terminator, 1.5 µL of 5× sequencing buffer, 3.2 pmol primer, and 1 µL of PCR product. Additional primers sets not covered by the RSS000010013 were also amplified and cycle sequenced as previously described.^30^ Excess BigDye terminators were removed using BigDye XTerminator, and samples were electrophoresed on the 3730 DNA Analyzer (Thermo Fisher Scientific) using the run module BDx_Rapid-Seq36_POP7 (Thermo Fisher Scientific). Sequence data were analyzed using the SeqScape software (Thermo Fisher Scientific) with GenBank *CFTR* genomic reference sequence NG_016465.

## Results

The CDC’s NSQAP sends DBS samples to participating laboratories engaged in CF newborn screening 4 times a year. Based on the information collected from the 63 laboratories that reported data for quarter 1 of 2016, the most commonly used method of DNA extractions was the Qiagen Generation DNA Purification and Elution Solution method (Table 1). This method is a relatively crude extraction that often results in a lower concentration of DNA. Thus, the Generation DNA extraction method was used in this study to validate the various genotyping and sequencing methods. The primary and secondary genotyping/sequencing methods reported by the laboratories for this quarter included 27 different methods that were either commercially available or laboratory developed. The number of mutations that each method detects is reported in Table 2 and ranged from 1 to 139 detected mutations for genotyping assays and 2 to an unlimited number of detected mutations for Sanger and next-generation sequencing methods.

DNA was extracted from a 3.2-mm punch taken from a DBS that contains approximately 3 mL of blood. Since newborn screening laboratories do not typically quantify DNA, a prescribed volume of extracted DNA was used in most of the assays rather than a set concentration or quantity. In order to better define the working range of DNA concentrations for the assays not commonly used in newborn screening laboratories, DNA extracts were quantified using real-time PCR, and the average quantity and range of concentrations are presented in Table 3.

All genotyping method results were compared with Sanger sequence data and found to have 100% concordance with the mutations included in their panels (Table 4 includes ACMG recommended mutations and variants, and Table 5 includes expanded panel mutations beyond the ACMG recommended). The CFTRdele2,3 (c.54–5940_273+10250del21kb) mutation detected by the xTAG CF60v2 kit was confirmed using the CF 139-variant assay kit because this mutation is not detectable using Sanger sequencing. The xTAG CF kits conditionally report the intron 9 poly T status (c.1210–12[5], c.1210–12[7], and c.1210–12[9]) when an R1 17H (c.350G>A) mutation is present, whereas these data can be seen for all samples using the InPlex 40+4 assay. Similarly, the F508C (c.1523T>G), I506V (c. 1516A>G), and I507V (c.1519A>G) variants are assayed, but results are only displayed when the software designates a “Mut D” call (indicating no normal sequence detected) for F508del (c.1521_1523delCTT) and/or I507del (c.1519_1521delATC) for the xTAG kits, but the F508C (c.1523T>G) is shown for all InPlex 40+4 samples. These variants when analyzed by the genotyping assays were 100% concordant with Sanger sequence data (Tables 4 and 5). For this study, only TaqMan Genotyping assays corresponding to the InPlex CF Molecular test 40+4 kit were assayed, however, additional *CFTR* mutation probe sets are available and can be used to create a more customized panel.

Three next-generation sequencing methods and 2 instrument platforms (MiSeqDx and Ion Torrent PGM) were used to characterize the CF DNA DBS repository. Both the MiSeqDx *CFTR* 139-variant genotyping assay and the AmpliSeq *CFTR* gene sequencing panel have a developed bioinformatics pipeline for analysis on their respective instruments, whereas the Accel-Amplicon *CFTR* panel, which is still in development, was analyzed using freeware bioinformatics tools.^26–29^ The results from all 3 next-generation sequencing methods were 100% concordant with Sanger sequence. Since Sanger sequencing cannot detect the CFTR dele2,3 mutation in the MiSeqDx *CFTR* 139-variant assay, this sample was compared with the xTAG CF60v2 and also found to be 100% concordant (Tables 4 and 5).

The MiSeqDx *CFTR* 139-variant next-generation sequencing assay which can accommodate up to 48 samples per flow cell had a density range between 473 ± 11 and 983 ± 9 K/mm^2^, with an average quality score ≥Q30 of 88.6% for read 1 and 79.9% for read 2. The Ion AmpliSeq *CFTR* libraries were pooled and run on six 318 chips; five 318 chips were loaded with 20 samples, and one 318 chip was loaded with 32 samples. The average mapped reads for the 5 chips with 20 samples was 276 939, and 97.2% of reads were on target with a read depth of 2 476 and a uniformity of 88.1%. The 318 chip with 32 samples had 102 862 mapped reads and 94.8% reads on target with a read depth of 705 and uniformity of 88.3%. The Ion AmpliSeq *CFTR* Panel was designed to distinguish the intron 9 PolyT 5 (c.1210–12[5]) from the 7 and 9T (c.1210–12[7] and c.1210–12[9]), so Poly T7 and 9 were not called using the bioinformatics pipeline. The AmpliSeq results were 100% concordant with Sanger sequence for all mutations and variants (Tables 4 and 5). In 1 sample, the Variant Caller plug-in did not make an automated call for an F508del/F508C (c.1521_1523delCTT/c.1523T>G) compound heterozygous sample. The F508del (c.1521_1523delCTT) was classified by the automated analysis as a no call, likely because of the complexity in the region when these mutations are present in the same sample. Examination of the sequence data did detect the presence of this mutation, and the correct genotype was called manually. In addition, an I507del/F508del (c.1519_1521delATC/c.1521_1523delCTT) sample required visual inspection for the final call for similar reasons. Since the results presented here are based on chemistry and algorithms from 2013, it is predicted that newer chemistries and algorithms may improve these calls.

The Accel-Amplicon *CFTR* panel libraries were run on the MiSeqDx in Research Run mode using 2 flow cells. A library of 94 samples was loaded on a micro flow cell and produced a density of 1299 ± 10 K/mm^2^ with a read quality of ≥Q30 of 88.8% for read 1 and 84.3% for read 2. A second library of 74 samples was loaded on a standard flow cell and produced a density of 698 ± 10 K/mm^2^ with read quality of ≥Q30 for 90.2% of read 1 and 80.4% for read 2. There was an average of 115 611 mapped reads with 87.2% of reads on target with a read depth of 1142 and 74.9% uniformity. Both FreeBayes and GATK^29^ was used to make variant calls because they produced different call frequencies. The output from both programs was used along with visualization of the data for analysis. As with the AmpliSeq assay, a sample containing I507del/F508del (c.1519_1521delATC/c.1521_1523delCTT) required visually inspection for the final call again due to the complexity in the region when these mutations are presented in the same sample.

In addition to the mutations listed in Tables 4 and 5,8 additional mutations in our CF DNA DBS repository that cannot be detected by any of the IVD genotyping assay were observed. These mutations can only be detected by Sanger and some of the next-generation sequencing methods. They include the following mutations: 124del23bp (c.-9_14del23), 185+4A>T (c.53+4A>T), F311del (c.933_935delCTT), 1288insTA (c.1153_1154dupTA), 2105–2117del13insAGAAA (c.1973_1985del13insAGAAA), L967S (c.2900T>C), M1101R (c.3302T>G), and S1235R (c.3705T>G). There was 100% concordance between all methods where the mutation was run, however, more complex mutations such as the 2105–2117del13insAGAAA(c.1973_1985del13insA-GAAA) had to be visually inspected and manually called for both the AmpiSeq and Accel-Amplicon assays, and the 124del23bp (c-9_14del23) was analyzed with no primer trimming for Accel-Amplicon (Note: only the normal sequence of this mutation was sequenced using the AmpliSeq assay in this study).

A summary of DNA quantity inputs, single-run capacity and assay time requirements, data analysis software, and mutation reports for each method is presented in Table 3. DNA quantity inputs are not reported for the xTAG CF and InPlex CF kits because DNA is not typically quantified prior to use in newborn screening laboratories, rather each run uses 5 µL of a 50-mL Generation DNA extraction from a 3.2-mm DBS punch. Library prep time, which is only reported for next-generation sequencing assays, includes PCR setup and run time, whereas PCR setup and run time is only reported for genotyping assays. Analysis time for Sanger sequencing and next-generation sequencing is not included in this table because it may vary depending on the software and pipeline utilized. The Open Array mentioned with TaqMan Genotyping was not used in this study, however, it is included in the table as it is an available option. All methods except Sanger sequencing were executed using DNA extracted from a 3.2-mm punch taken from a DBS using the Generation DNA extraction method.

## Discussion

All US states and many international laboratories screen their newborn population for CF with the majority using second-tier CFTR mutation detection assay as part of their screening algorithm. Although these programs have been very effective,^7,8^ CF newborn screening has a low-positive predictive value with >90% false-positives. The reason for the low-positive predictive value is that most programs use a panel of only 23 to 40 CF-causing mutations, and a screen-positive sample only has to contain 1 *CFTR* mutation.^12,18,31^ As CF is a recessive disease, carriers of a single *CFTR* mutation are initially flagged as false-positives. In order to increase the positive predictive value of the CF screen to reduce the burden on the CF care centers, some newborn screening programs are exploring more comprehensive mutation detection assays with the goal of a screen positive being defined as babies with elevated IRT and 2 CF-causing mutations. A 2-mutation detection strategy could increase the false-negative rate if the panel of *CFTR* mutations was not sufficiently large enough to address the spectrum of mutations across diverse ethnic populations. Using variants identified by CFTR2 project,^32^ a study by Baker et al found that a panel of 162 mutations was not sufficiently comprehensive to capture all babies identified by the current algorithm in Wisconsin.^18^ Expanding the CFTR2 panel of mutations to 276 mutations and variants, 2 babies with known mutations in the Wisconsin study would still have been reported as false negatives.^33^ With an increasingly diverse ethnic population occurring in the most US state populations, it is expected that a predefined expanded panel of mutations approach will likely be insufficient to define a screen positive as 2 *CFTR* mutations, resulting in the continued high false-positive rates.

To address the limitations of genotyping panels, gene sequencing methods enable every base within the *CFTR* gene to be screened. Currently, there are 2 distinct sequencing methodologies used. The older, more established method uses Sanger sequencing and is currently being used for newborn screening in the US state of California.^34^ Next-generation sequencing is the second and more recent technology that enables more comprehensive and higher throughput screening of CF samples. Although these approaches solve the issue of being able to identify uncommon mutations particularly in minority populations,^34^ it creates a new issue, which is the identification of babies who do not have CF but rather CRMS.^35^ The Cystic Fibrosis Foundation describes CRMS as infants with hypertryp-sinogenemia on newborn screening who have sweat chloride values <60 mmol/L and up to 2 CFTR mutations, at least 1 of which is not clearly categorized as CF causing.^36^

This study demonstrates that NSQAP’s CF DNA DBS repository is appropriate for use with CF screening assays as they are performed today both in the United States and internationally. These repository samples would also support next-generation sequencing assays for *CFTR* if programs choose to modify their screening algorithms to require 2 *CFTR* mutations. The input DNA is a critical component to all molecular tests and the DNA extraction methods reported from NSQAP participants range from a very crude methanol boil preparation to a more purified extraction involving column purification. The majority of US programs use a commercially available simple purification that involves wash steps followed by a boil step (Table 1). In addition, the CF DNA DBS repository samples used in this study have a lower DNA yield than newborn DBS because they were made from adult blood that has a lower average white blood cell count than newborns (7.4 × 10^6^ per mL of blood vs 1.9 × 10^7^ per mL of blood, respectively).^37^

All of the genotyping and sequencing methods tested on the repository samples provided robust and accurate results using the crude DNA extraction and is consistent with previous studies involving next-generation *CFTR* analysis using newborn DBS.^11,38-40^ Currently, the most commonly used genotyping assays in the United States, xTAG CF39v2 and InPlex CF 40+4, have defined mutation panels of 39 and 40 mutations, respectively. Since the InPlex CF 40+4 will be discontinued in early 2016 (note that the InPlex CF 23 will continue to be available), we also tested a custom TaqMan panel of mutations that mirrors the *CFTR* mutations in the InPlex CF 40+4. The TaqMan approach is different from the other genotyping approaches in that each mutation is a separate assay allowing for the easy addition or deletion of mutations. This approach requires more input DNA if it is performed in a 384-well format as presented here. There is a higher throughput version available for the OpenArray platform, however, it was not tested in this study. The next-generation sequencing approaches used in this study were selected to test the utility and robustness of crude low-concentration DNA with different next-generation sequencing platforms and library preparation assays. Although the US Food and Drug Administration (FDA)-approved CF 139-Variant Assay uses next-generation sequencing technology, it reports a genotype for a defined panel of mutations and variants. The mutation panel is not diverse enough to define a positive newborn screen positive as having 2 *CFTR* mutations; Illumina does offer an FDA-approved *CFTR* gene sequencing assay that was not tested in this study. Given the similar technology and work flow, it is anticipated that this method would also work well with DNA extracted from DBS. The *CFTR* gene next-generation sequencing assays tested in this study were the AmpliSeq *CFTR* Community Panel on the Ion Torrent PGM and the Accel-Amplicon *CFTR* Panel on the MiSeq. The Accel-Amplicon *CFTR* Panel can also be made for use with the Ion Torrent PGM.

The general trend of increasing population diversity, new technology introductions allowing for expanded mutation screening or gene sequencing, and the varying sizes of newborn screening programs are just 3 of many factors contributing to the complexities of newborn screening for CF. In addition, the current newborn screening algorithms have a high false-positive rate, prompting some programs to consider whether the definition of a CF screen positive should be redefined. The NSQAP offers quality assurance tools and services to newborn screening laboratories as they explore transitioning from one technology to another to meet their changing needs. The CF DNA DBS repository provides laboratories with representative samples with rare *CFTR* mutations for robust testing and evaluation purposes. The study presented here is a comprehensive characterization of these DBS samples and highlights their utility for a diverse range of methods being used in CF newborn screening today as well as next-generation sequencing assays that can be used in the future.

## Figures and Tables

**Table 1:** DNA Extration Methods Used by the 2016 Quarter 1 CF DNA PT Participants.

DNA Extraction Methods Used by CF DNA PT Participants	No. of Laboratories
Qiagen QIAamp spin columns (manual or robotic)	5
Qiagen magnetic bead kit (EZ1 or BioSprint 96)	2
Qiagen Generation DNA purification and DNA elution solutions	22
Sigma Aldrich Extract-N-Amp	3
In-house alkaline lysis prep	7
In-house boiling prep	6
In-house lysis boiling prep	1
Other	11
No response	6

Abbreviation: CF, cystic fibrosis.

**Table 2:** Primary and Secondary Genotyping Methods and Number of Mutations/Variants Detected by each Method for the 2016 Quarter 1 CF DNA PT Participants.

	Labs using Genotyping/ Sequencing Method				Mutations/Variants Detected by Genotyping/ Sequencing Methods				
Genotyping/Sequencing Methods	# labs using as primary method	# labs using as secondary method	U.S. labs	non-U.S. labs	# mutations detected	# ACMG mutations	# expanded mutations	variants (F508C, 1506V, 1507V)	Intron 9 (5/7/9T)
Hologic CF InPlex^®^ Molecular Test—ACMG	3	1	4	0	23	23	1^a^	F508C	5/7/9T
Hologic CF InPlex^®^ Molecular Test 40+4	20	6	19	1	40^b^	23	19	F508C	5/7/9T
Luminex Molecular Diagnostics xTAG^®^ CF—ACMG only	0	0	0	0	23	23	0	Yes	5/7/9T
Luminex Molecular Diagnostics IVD xTAG^®^ CF39 v2	7	4	5	2	39	23	16	Yes	5/7/9T
Luminex Molecular Diagnostics xTAG^®^ CF60 v2	1	0	1	0	60	23	37	Yes	5/7/9T
Luminex Molecular Diagnostics xTAG^®^ CF7I v2	0	0	0	0	71	23	48	Yes	5/7/9T
Luminex Platform and Laboratory Developed Test	1	0	1	0	40	15	25	No	No
Elucigene Diagnostics Elucigene^®^ CF4v2	1	0	0	1	4	4	0	No	No
Elucigene Diagnostics Elucigene^®^ CF30v2	3	0	0	3	29	19	10	No	No
Elucigene Diagnostics Elucigene^®^ CFEU2vl	1	1	0	1	51	23	27	No	5/7/9T
Abbott Molecular CF Genotyping Assay v3	2	1	0	4	32	23	9	Yes	5/7/9T
Fujirebio INNO-LiPA^®^ Strip 19	0	1	0	1	19	12	7	No	No
Fujirebio INNO-LiPA^®^ Strips 17+19	3	2	0	3	36	23	13	No	5/7/9T
Sequenom^®^ assays other than HerediT™ CF (MALDI-TOF Mass Spectrometry)	1	1	0	3	12–42	1 1–21	1–21	No	No
ViennaLab Diagnostics GmbH CF StripAssay^®^	1	0	0	1	34	23	1 1	No	5/7/9T
Allele-specific Oligonucleotide PCR	2	0	1	1	1–9	1–9	0	No	No
High Resolution Melt Technology	2	0	0	2	3–11	3–8	0–3	Unknown	No
Real-time PCR Allelic Discrimination Assay (ie TaqMan^®^)	2	0	2	0	1	1	0	No	No
In-house Amplification Refractory Mutation System	1	1	0	1	1	1	0	No	No
In-house single nucleotide primer extension assay	1	0	0	1	12	10	1	No	5/7/9T
PCR/Heteroduplex Analysis/Gel Electrophoresis	0	2	0	2	1	0	1	No	No
Capillary Electrophoresis	0	1	0	1	3	3	0	No	No
Amplification and Restriction Fragment Length Polymorphism Analysis (PCR-RFLP)	2	1	0	2	5–9	4–8	1-1	No	No
Amplification and Polyacrylamide Gel Electrophoresis (PCR-PAGE)	0	0	0	1	1	1	0	excludes variants	No
Next-generation sequencing—lllumina MiSeqDx™ Cystic Fibrosis 139 Variant Assay	1	0	0	1	139	23	113	Yes	5/7/9T
Next-generation sequencing—Multiplicom Molecular Diagnostics CFTR MASTR™ v2	2	0	0	1	varies^c^	20–23^c^	varies^c^	varies^c^	varies^c^
Next-generation sequencing—Ion AmpliSeq™ CFTR Community Panel	0	1	0	1	varies^c^	23	varies^c^	Yes	5T
All other gene sequencing protocols including Sanger and Next Gen	5	5	1	8	varies^c^	2–23	varies^c^	Yes^c^	5/7/9T^d^
Other—Hydrolysis probe	1	0	0	1	4	4	0	No	No
Other—LiGHT SNiP	0	1	0	1	7	7	0	No	No

a The 2183AA>G mutation is used for the interpretation of the 2l84delA mutation and is not reported.

b Note that the InPlex 40+4 contains two non CF causing variants, 1148T (c.443T>C) and D1270N (c.3808G>A) are not counted in these numbers.

c Varies by the sequencing technology used and/or whether filters are applied to mask certain results.

d Intron 9 - 5/7/9T detetable by Sanger if included in assay.

**Table 3:** Comparison of Method Input DNA Required, Mutations Detected, Number of Reactions, Run Time, and Run Capacity.^a^

Method	No. Mutations/ Variants Detected	No. Reactions	DNA Volume Used in Study^b^	Avgerage DNA Qty Used Per Reaction (Min- Max)^b^	Library Prep Time	PCR Setup and Run Time	Post-PCR Processing/ Run Time	Instrument Run Time	Total Run Time	Single-Run Capacity^c^	System Dedi- cated Software	Mutation Report
Sanger	Unlimited in regions amplified	42PCR rxns86 cycle sequencing	42 µL(I µL/ PCR rxn)	5–10 ng	NA	3 hours	4 hours	14.5 hours	21.5 hours	Nine 384-well plates (+2, 96-well plates with varying run conditions)	Y SeqScape	Does not distinguish variants from defined mutations—requires expert interpretation
Ion AmpliSeq CFTR Community Panel	Unlimited in regions amplified	2 Pools	12 µL (6 µL/ pool)	18.2 ng (0.76–8.98 ng/µL)	4–6 hours	NA	6–7 hours	2–7 hours (dependent on chip size)	12–20 hours	Eight 314 chip (500 ×) Forty-nine 316 chip (500 ×) Ninety-six 318 chip (500 ×)	Y Coverage Analysis Variant Caller	CFTR2 defined mutations and variants listed in Hotspot file are annotated (Legacy and HGVS)—novel mutations and variants require expert interpretation
Swift Biosciences Accel- Amplicon CFTR Panel	Unlimited in regions amplified	1 Pool	5–10 µL	19.3 ng (0.55– 5.21 ng/µL)	2.5 hours	NA	1.5 hours	28 hours	32 hours	Forty-eight Nano flow cell (400×) Forty-eight Micro flow cell (1600×)	N^d^ Freeware: Cutadapt, BWA-MEM, FreeBayes, GATK	Does not distinguish variants from defined mutations—requires expert interpretation
MiSeqDx Cystic Fibrosis 139-Variant Assay	139	1 Pool	5 µL	8.95 ng (0.55– 3.88 ng/µL)	5 hours	NA	3 hours	28 hours	36 hours	Forty-eight flow cells	Y MiSeq Reporter	Defined mutations identified
Hologic InPlex CF Molecular Test 40+4	42 + 2 variants	1 rxn	5 µL	Not quantified	NA	2.5 hours	1 hour	5 minutes	3.5 hours	8 Cards	Y Call Reporting Software	Defined mutations identified
Luminex xTAG CF39v2 kit	39+4 variants	1 rxn	5 µL	Not quantified	NA	2 hours	3.5 hours	1 hour	6.5 hours	Ninety-six 96- well plates	Y TDAS CFTR	Defined mutations identified
Luminex xTAG CF60v2 kit	60+4 variants	1 PCR rxns 2 ASPE rxns	5 µL	Not quantified	NA	2 hours	4 hours	1 hour	7 hours	Forty-eight 96- well plates	Y TDAS CFTR	Defined mutations identified
Thermo Fisher Scientific TaqMan SNP Genotyping	44	45 rxns	45 µL^e^ (1 µL/mutation)	1.59 ng (0.62– 4.82 ng/µL)	NA	15 minutes - 96-well plate 30 minutes - 384-well plate 30 minutes - OpenArray plate	NA	1.5 hours	~2 hours - 96- well plate ~2 hours - 384-well plate ~4 hours - OpenArray plate	Two 96-well plates Eight 384- well plate Forty-eight OpenArray plates (64 assay format)	Y Genotyper	Defined mutations identified—some mutations require interpretation using multiple probes (intron 9-5/7/9T and I507del and F508del region)

Abbreviations: ASPE, allele specific primer extension; CF, cystic fibrosis; CFTR, CF transmembrane regulator; DBS, dried blood specimen; HGVS, human genome variation society; NA, not available; NBS, newborn screening; PCR, polymerase chain reaction; rxn, reaction; SNP, single-nucleotide polymorphism.

a The run times in this table are based on our laboratory’s experience, however it does not include analysis of the data. The Bioinformatics are described in the materials and methods.

b Typically DNA is not quantitated prior to use in NBS labs, so some of the quantities are estimated based on average known concentration of DNA extracted from adult DBS.

c The single-run capacity indicates the number of samples that can be loaded onto an instrument at a time.

d The Accel-Amplicon CFTR panel is still in development, the company plans on offering a Bioinformatic pipeline (T. Harkins, personal communication, March 29, 2016).

e OpenArray was not performed in this study. The manufacturer recommended input is 2.5 µL of DNA exctracted from DBS.

**Table 4:** ACMG Recommended Mutations found in the CF DNA DBS Repository Samples as Characterized by Next-Generation Sequencing and Mutation Analysis.

Mutation (Legacy Name)	Mutation (HGVS)	Sanger	AmpliSeq CFTR Community Panel	Accel- Amplicon CFTR Panel	CF 139- Variant Assay	InPlex CF 40+4	xTAG CF39v2 kit	xTAG CF60v2 kit	TaqMan SNP Genotyping
**F508del**	**c.1521_1523delCTT**	+	+	+	+	+	+	+	+
**I507del**	**c.1519_1521delATC**	+	+	+	+	+	+	+	+
**G542X**	**c.1624G>T**	+	+	+	+	+	+	+	+
**G85E**	**c.254G>A**	+	+	+	+	+	+	+	+
**R117H**	**c.350G>A**	+	+	+	+	+	+	+	+
**621 + 1G>T**	**c.489+ 1G>T**	+	+	+	+	+	+	+	+
**711 + 1G->T**	**c.579+ 1G>T**	+	+	+	+	+	+	+	+
**R334W**	**c.1000C>T**	+	+	+	+	+	+	+	+
**R347P**	**c.1040G>C**	+	+	+	+	+	+	+	+
**A455E**	**c.1364C>A**	+	+	+	+	+	+	+	+
**1717–1G>A**	**c.1585+ 1G>A**	+	+	+	+	+	+	+	+
**R560T**	**c.1679G>C**	+	+	+	+	+	+	+	+
**R553X**	**c.1657C>T**	+	+	+	+	+	+	+	+
**G551D**	**c.1652G>A**	+	+	+	+	+	+	+	+
**1898+ 1G>A**	**c.1766+ 1G>A**	+	+	+	+	+	+	+	+
**2184delA**	**c.2052delA**	+	+*	+	+	+	+	+	+
**2789+5G>A**	**c.2657 + 5G>A**	+	+	+	+	+	+	+	+
**3120+ 1G>A**	**c.2988+ 1G>A**	+	+	+	+	+	+	+	+
**R1162X**	**c.3484C>T**	+	+	+	+	+	+	+	+
**3659delC**	**c.3528delC**	+	+	+	+	+	+	+	+
**3849+ 10kbC>T**	**c.3717+ 12191C>T**	+	+	+	+	+	+	+	+
**W1282X**	**c.3846G>A**	+	+	+	+	+	+	+	+
**N1303K**	**c.3909C>G**	+	+	+	+	+	+	+	+
F508C	c.1523T>G	+	+	+	nr	+	nr	nr	+
T5	c.1210–12[5]	+	+	+	CR	+	CR	CR	+
T7	c.1210–12[7]	+	ND	+	CR	+	CR	CR	+
T9	c.1210–12[9]	+	ND	+	CR	+	CR	CR	+

Abbreviations: ACMG, American College of Medical Genetics; CF, cystic fibrosis; CFTR, CF transmembrane regulator; CR, conditionally reported with an R117H present; ND, not distinguishable; nr, not reported but used for correct mutation interpretation; SNP, single-nucleotide polymorphism;

+, mutations detected;

+*, not assayed in this study but detectable by method.

a Boldface entries indicates ACMG mutations.

**Table 5:** Mutations Excluding ACMG Recommended Mutations Found in the CF DNA DBS Repository Samples as Characterized by Next-Generation Sequencing and Mutation Analysis.

Mutation (Legacy Name)	Mutation (HGVS)	Sanger	AmpliSeq CFTR Community Panel	Accel- Amplicon CFTR Panel	CF 139- Variant Assay	InPlex CF 40+4	xTAG CF39v2 kit	xTAG CF60v2 kit	TaqMan SNP Genotyping
394delTT	c.262_263delTT	+	+*	+	+	+	+	+	+
A559T	c.1675G>A	+	+	+	+	NA	+	+	AV
S549N	c.1646G>A	+	+	+	+	+	+	+	+
2183AA>G	c.2051_2052delAAinsG	+	+	+	+	+	+	+	+
2307insA	c.2175_2176insA	+	+*	+	+	+	+	+	AV
Y1092X	c.3276C>A or c.3276C>G	+	+	+	+	+	+	+	+
3876delA	c.3744delA	+	+	+	+	+	+	+	+
3905insT	c.3773dupT	+	+	+	+	+	+	+	+
CFTR dele2,3	c.54- 5940_273+10250del21kb	NA	NA	NA	+	NA	NA	+	AV
E60X	c.178G>T	+	+	+	+	+	NA	+	+
R75X	c.223C>T	+	+	+	+	NA	NA	+	AV
406–1G>A	c.274–1G>A	+	+*	+	+	NA	NA	+	AV
L206W	c.617T>G	+	+	+	+	NA	NA	+	AV
935delA	c.803delA	+	+*	+	NA	NA	NA	+	AV
Q493X	c.1477C>T	+	+	+	+	+	NA	+	+
Q890X	c.2668C>T	+	+	+	+	NA	NA	+	AV
1677delTA	c.1545_1546delTA	+	+	+	+	NA	NA	+	AV
2055del9>A	c.1923_1931del9insA	+	+	+	NA	NA	NA	+	AV
R1158X	c.3472C>T	+	+	+	+	NA	NA	+	AV
R1066C	c.3196C>T	+	+	+	+	NA	+	+	AV
W1089X	c.3266G>A	+	+	+	+	NA	NA	+	AV
D1152H	c.3454G>C	+	+	+	NA	+	NA	+	+
3791delC	c.3659delC	+	+*	+	+	NA	NA	+	AV
D1270N	c.3808G>A	+	+	+	NA	+	NA	NA	NA
Q39X	c.115C>T	+	+*	+	+	NA	NA	NA	AV
663delT	c.531delT	+	+	+	+	NA	NA	NA	AV
P205S	c.613C>T	+	+	+	+	NA	NA	NA	AV
1154 insTC	c.1022_1023insTC	+	+*	+	+	NA	NA	NA	AV
1248+1G- >A	c.1116+1G>A	+	+	+	+	NA	NA	NA	AV
L467P	c.1400T>C	+	+*	+	+	NA	NA	NA	AV
S492F	c.1475C>T	+	+	+	+	NA	NA	NA	AV
1812–1G>A	c.1680–1G>A	+	+	+	+	NA	NA	NA	AV
2184insA	c.2052dupA	+	+	+	+	NA	NA	NA	NA
3121–1G- >A	c.2989–1G>A	+	+	+	+	NA	NA	NA	AV
3272– 26A>G	c.3140–26A>G	+	+	+	+	NA	NA	NA	AV
R1066H	c.3197G>A	+	+	+	+	NA	NA	NA	AV
W1204X	c.2611G>A or c.3612G>A	+	+	+	+	NA	NA	NA	AV
G1244E	c.3731G>A	+	+*	+	+	NA	NA	NA	AV

Abbreviations: AV, assay available but not evaluated in this study; CF, cystic fibrosis; CFTR, CF transmembrane regulator; NA, not available in assay; SNP, single-nucleotide polymorphism;

+, mutations detected;

+*, specimen containing mutation not assayed but normal region sequenced in this study.
